# The Interaction Analysis of SNP Variants and DNA Methylation Identifies Novel Methylated Pathogenesis Genes in Congenital Heart Diseases

**DOI:** 10.3389/fcell.2021.665514

**Published:** 2021-05-04

**Authors:** Jing Wang, Xiaoqin Ma, Qi Zhang, Yinghui Chen, Dan Wu, Pengjun Zhao, Yu Yu

**Affiliations:** ^1^Department of Pediatric, Yangpu District Shidong Hospital, Shanghai, China; ^2^Institute for Developmental and Regenerative Cardiovascular Medicine, Xinhua Hospital, School of Medicine, Shanghai Jiao Tong University, Shanghai, China; ^3^Department of Pediatric Cardiology, Xinhua Hospital, Shanghai Jiao Tong University School of Medicine, Shanghai, China; ^4^Shanghai Children’s Medical Center, Shanghai Jiao Tong University School of Medicine, Shanghai, China

**Keywords:** congenital heart defects, single-nucleotide polymorphisms, DNA methylation, cpG island, meQTLs

## Abstract

Congenital heart defect (CHD) is a rare and complicated disease with a high mortality rate. Its etiology remains unclear and includes many aspects. DNA methylation has been indicated to be involved in heart development in the early stage of life, and aberrant methylation level was related to CHDs. This study provides the first evidence of the cross talk of SNP variants and DNA methylation in clarifying CHD underlying genomic cause. We gathered whole exome sequencing (WES) data for Group 1 consisting of patients with PA (*n* = 78), TOF (*n* = 20), TAPVC (*n* = 78), and PDA (*n* = 40), and 100 healthy children as control group. Rare non-synonymous mutations and novel genes were found and highlighted. Meanwhile, we carried out the second analysis of DNA methylation data from patients with PA (*n* = 3), TAPVC (*n* = 3), TOF (*n* = 3), and PDA (*n* = 2), and five healthy controls using 850 K array in Group 2. DNA methylation was linked to WES data, and we explored an obvious overlap of hyper/hypomethylated genes. Next, we identified some candidate genes by Fisher’s exact test and Burden analysis; then, those methylated genes were figured out by the criteria of the mutation located in the CpG islands of the genome, differential methylation sites (DMS), and DNA methylation quantitative trait loci (meQTLs) in the database, respectively. Also, the interaction of differentially methylated candidate genes with known CHD pathogenetic genes was depicted in a molecular network. Taken together, our findings show that nine novel genes (ANGPTL4, VEGFA, PAX3, MUC4, HLA-DRB1, TJP2, BCR, PKD1, and HK2) in methylation level are critical to CHD and reveal a new insight into the molecular pathogenesis of CHD.

## Introduction

Congenital heart defect (CHD) is the most common human birth defect, accounting for 0.8% of live infants (Bouma and Mulder, 2017; Tennant et al., 2010). It contains various types of heart diseases, such as patent ductus arteriosus (PDA), tetralogy of Fallot (TOF), total anomalous pulmonary venous connection (TAPVC), and pulmonary atresia (PA), which encompass great vascular abnormality in heart structure. PDA is a congenital shunt vessel, connecting the proximal descending aorta to the roof of the pulmonary artery. The incidence of PDA accounts for 0.03–0.08% of live births among term infants (Kumar et al., 2019). TOF is the most common form of cyanotic CHD with an incidence of approximately four in 10,000 live births, and the anatomical characteristics include ventricular septal defect (VSD), pulmonary stenosis (PS), right ventricular hypertrophy, and an overriding aorta (Morgenthau and Frishman, 2018). TAPVC is a rare congenital cardiac anomaly characteristic of pulmonary veins directly connecting to the right atrium or systemic venous system (Acevedo et al., 2017).

The etiology of cardiac defects includes complex aspects, environmental factors, gene variations, and epigenetic changes, in which DNA methylation is best explored. The progress of DNA sequencing technology reveals that genetic variation plays an increasingly critical role in the causes of CHD (Dorn et al., 2014; Page et al., 2019). Despite the diversity of the CHD category, whole exome sequencing (WES) can identify a series of potentially causal genes and elucidate shared pathways critical to cardiac development. In addition to the important effects of the variants, DNA methylation is suggested to be involved in the heart development of mammalian animals (Serra-Juhé et al., 2015). DNA methylation is the most widely explored epigenetic modification, which occurs in the context of CpG dinucleotides (Jones et al., 2015). Methylation is involved in the occurrence of various diseases, and researchers have proved that aberrant DNA methylation was associated with the incidence of CHD. For TOF, researchers have proved that a large number of promoters carry DNA hyper- or hypomethylation genes related to cell proliferation, embryonic development included (Gong et al., 2019). NOTCH1 was identified as the major cardiac damaging gene, and there were methylation changes in the NOTCH pathway, such as IDB4 (Greenway et al., 2009). Additionally, some studies revealed that both gene mutations and methylation modifications account for the development of CHD, such as CITED2 mutations along with promoter region methylation (Xu et al., 2014; Sheng et al., 2016).

Interestingly, the dysregulation of DNA methylation during stages of embryonic development may lead to inappropriate silencing of gene expression, thereby increasing the risk of cardiac malformation (Grunert et al., 2016). In another study, hypermethylation was observed in the key cardiac signaling pathway factors Nkx2.5 and Hand1, in patients diagnosed as TOF and VSD. Expectedly, the expression of the two factors was proved to be downregulated (Sheng et al., 2013).

Considering the complexity of cardiogenesis, focusing on single gene or gene mutations is not beneficial to understand its etiology. The methylation study in CHD is still novel and could prompt the advancement of cardiac defect study. Importantly, the interaction of SNP variants and DNA methylation, accompanying the changes of gene expression in CHD occurrence, remains largely unknown. Therefore, we carried out a comprehensive analysis of whole genome DNA methylation study on blood samples of TOF, PA, TAPVC, PDA, and normal samples, and combined the summary of WES data. Our research evaluated the overlapping roles of DNA methylation and gene mutations in CHD and discussed their potential relationship.

## Materials and Methods

### Study Population

Group 1 included 216 unrelated patients for WES analysis, which consisted of 78 TAPVC, 78 PA/VSD patients, 40 PDA patients, and 20 TOF patients. Data of WES analysis were derived from our previous studies (Chen et al., 2020; Shi et al., 2020; Shi et al., 2018). Group 2 contained 16 cases (three TAPVC, two PDA, three TOF, three PA, and five healthy children); blood samples were collected, checked, standardized, and analyzed for DNA methylation. All subjects in Group 2 were recruited *via* the Department of Pediatrics, Yangpu District Shidong Hospital. The study was carried out according to the Declaration of Helsinki, and the method applied to collect human samples of blood was approved by the Ethics Committee of the Department of Pediatrics, Yangpu District Shidong Hospital. All participants or their guardians have signed the written informed consent before the study. Clinical information is shown in Supplementary Table 1.

### WES Assay

Whole exome sequencing was applied for sequencing in the first group. WES was carried out using the Agilent Sure Select Target Enrichment kit (V6 58 Mb; Agilent Technologies) for sequence capture and the Illumina HiSeq2500 for sequencing (Illumina) to a target depth of 100 (Chen et al., 2020; Shi et al., 2020; Shi et al., 2018).

### DNA Preparation for Methylation Detection

Genomic DNA was extracted from human blood employing the QIAampTM DNA and Blood Mini kit (Qiagen) referring to the manufacturer’s protocol. DNA concentration and integrity were assessed by a NanoDrop 2000 spectrophotometer (Thermo Fisher Scientific, Waltham, MA, United States) and agarose gel electrophoresis, respectively. DNA was bisulfite treated using the Zymo Research EZ DNA methylation Gold Kits (Zymo Research, Irvine, CA, United States).

### Genome-Wide DNA Methylation Data Analysis

Bisulfite-converted DNA was analyzed on an Illumina Infinium Methylation EPIC 850 K Bead Chip (Illumina) (Kommoss et al., 2020). We extracted microarray data and figured out DNA methylation level through GenomeStudio Methylation Module v1.8 software (Version 2011.1) with default parameters. We used the normalized data to compute the DNA methylation levels, displaying β values ranging from 0 to 1, corresponding to unmethylated and methylated sites, separately. We performed cluster analysis of differentially methylated sites and analyzed biological function.

### Differential Methylation Sites Screening

The correlation of gene methylation levels between samples is an indicator for testing the reliability of experiments and the rationality of sample selection. The correlation calculation data come from the standardized Beta value using Pearson.

Differential methylation sites (DMS) were analyzed and screening criteria of DMS were as follows:

1. The absolute value of “deltaBeta” is greater than 0.1;
2. “*P* value” < 0.01 (for biological duplication),

where *P* value is the calculation result of the limma package, and deltaBeta is an index that measures the degree of methylation difference between the case group and the healthy group. The calculation formula is as follows:

deltaBeta = Beta case − Beta control

### Methylated Genes Selection

Three ways were mainly considered between the mutation and methylation:

Firstly, the mutations in WES data of 216 patients screened by Fisher’s exact test and Burden analysis have differential DNA methylation.

Secondly, the sites of mutation-related genes filtered by Fisher’s exact test and Burden analysis were located in the CpG island of the genome.

Thirdly, according to the data of DNA methylation quantitative trait loci (meQTLs^1^) in the tumor (the relationship between the mutation site and DNA methylation; it has been analyzed that there is a regulatory relationship in the tumor), the mutation site/gene and differential methylation were established. This result contains two parts of *cis* and *trans* regulation (close regulation of methylation and long-range regulation of methylation) (Gamazon et al., 2013; Li et al., 2013).

### Gene Ontology and the Kyoto Encyclopedia of Genes and Genomes Description

Gene ontology (GO^2^) and the Kyoto Encyclopedia of Genes and Genomes (KEGG^3^) pathway enrichment analyses were carried out using the scripts in Python to demonstrate the functional and biological pathways of DMS or candidate methylated genes figured out according to the above three ways. GO terms and KEGG with *P* < 0.05 were considered obviously enriched by differential methylation locus−related genes.

### Protein–Protein Interaction Analysis

The Search Tool for the Retrieval of Interacting Genes database was applied for the PPI analysis^4^, which could assess and integrate direct (physical) and indirect (functional) associations. The gene set consisted of either CHD-causing genes or the statistically significant CHD-related methylated genes and were positioned to the PPI network altogether. The gene set was provided in Supplementary Material 1.

### Gene Expression Analysis of the Microarray Datasets

Previously, we obtained the expression data in homo early embryonic heart at different stages (Carnegie stages 10–16) after medical termination of pregnancy measured with the Affymetrix HTA 2.0 microarray platform (Chen et al., 2020; Shi et al., 2020; Shi et al., 2018). The expression levels of our methylated candidate genes in human embryonic heart were shown using the median value.

### Statistical Analysis

Statistical differences between the CHD and the control group were compared by the Fisher’s exact test mode. The obvious difference of methylation loci set standards at a threshold of | Delta_ Beta value| > 0.1 and *P* value < 0.01 (The Delta_Beta value is calculated by the difference of Avg_Beta between the control and the CHD group, which means the difference of methylation at each site).

## Results

### CHD-Associated Differential SNPs Captured by Fisher’s Exact Test and Burden Analysis

**FIGURE 1:**
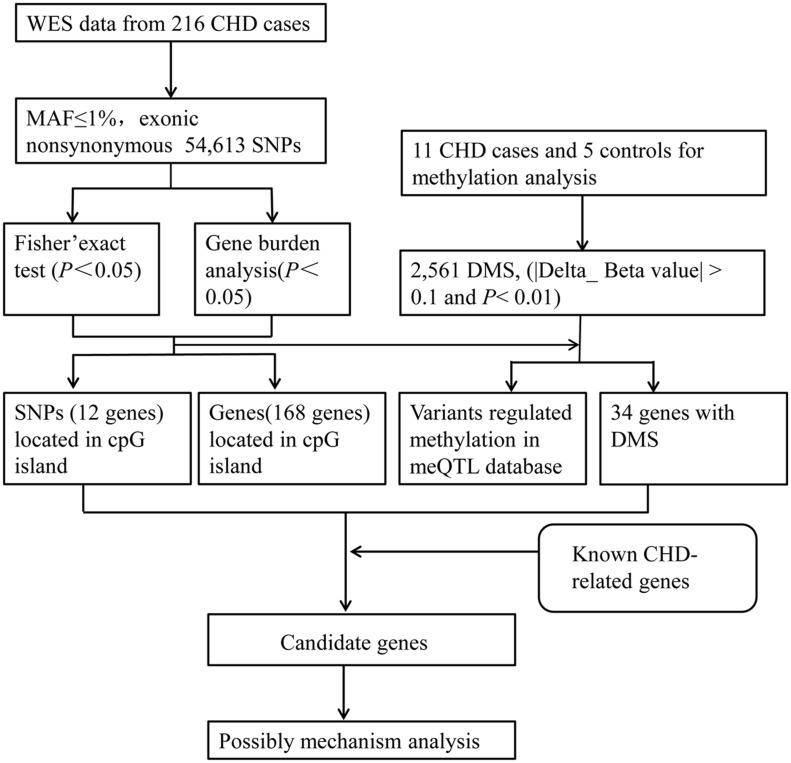
The analytical strategy workflow for candidate gene filtration. A schematic overview of the different steps taken during DNA methylation sequencing and next-generation sequencing analysis in CHD patients. Through Fisher’s exact test and Burden analysis, candidate genes were identified and then figured out by the criteria of mutation located in the CpG islands of the genome, differential methylation sites, and DNA methylation quantitative trait loci (meQTLs) in the database. SNP, single-nucleotide polymorphism; WES, whole-exome sequencing; MAF, minor allele frequency; DMS, differential methylation sites.

**FIGURE 2:**
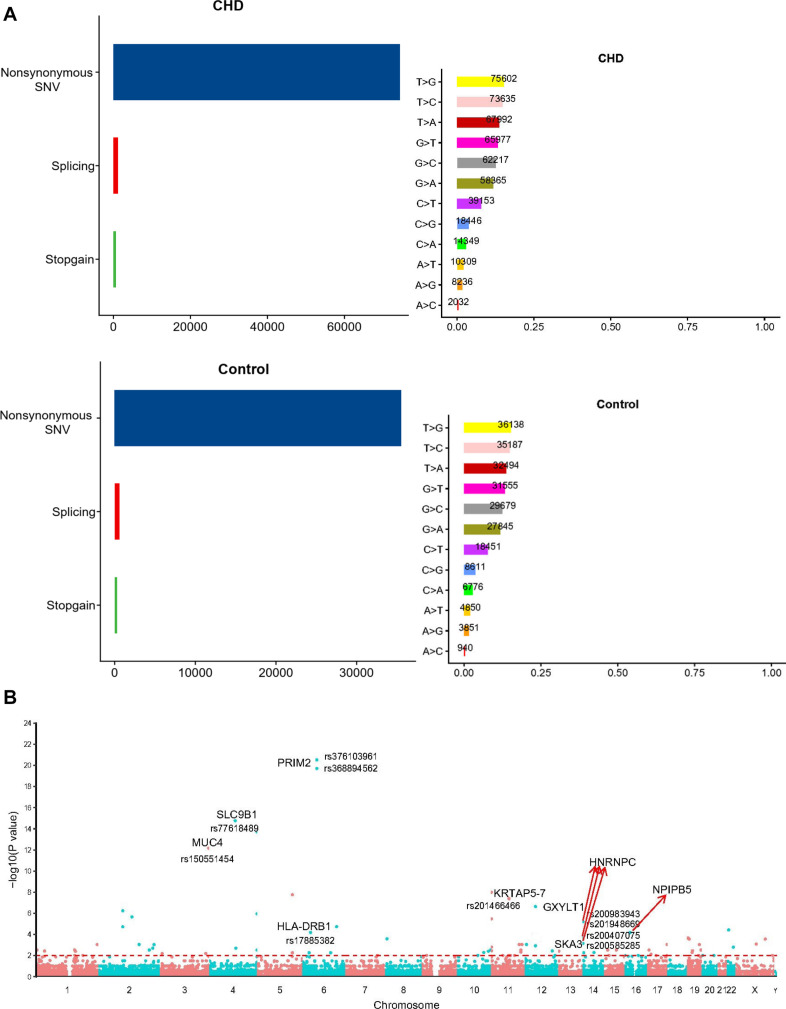
The comparison of the rare damaging variants between the case and control groups. **(A)** The number of variants in each variant classification and variant type was presented. **(B)** The Manhattan plot of CHD-associated variants filtrated by Fisher’s exact test. Each node represented a variant, and the *y*-axis represented the statistical significance level. The top nine genes with higher mutation frequency were displayed in the picture (*P* < 0.01).

Firstly, we put forward an analytical flow chart to present our study clearly (Figure 1). Then, to tested the pathogenic SNP candidates, we identified WES data by statistical comparisons from 216 cases and 100 controls in Group 1. We found 54,613 function variants with SIFT damaging by Fisher’s exact test (MAF < 0.05). The non-synonymous SNV occupied the most variant types in both case and control groups (Figure 2A). T > G change also accounted for most of the base mutations than any other sorts in these two groups. Moreover, 340 genes through Fisher’s exact test (*P* < 0.05) and 404 genes with Burden analysis were found based on the 54,613 rare damaging variants consistently. We observed nine genes with the top potential pathogenicity with thresholds of 0.01 for *P* value, and the incidence frequency of these genes in the case group was higher than that in the control group (Figure 2B). Therefore, HLA-DRB1, PRIM2, SLC9B1, MUC4, NPIPB5, KRTAP5-7, GXYLT1, HNRNPC, and SKA3 were considered as the ones with higher frequency. In particular, variants fell from the genes of MUC4 and HLA-DRB1, which showed a predominant significance (*P* value < 2e4) and suggested that the two genes played important roles in the CHD pathogenesis.

### CHD-Associated DMS

**FIGURE 3:**
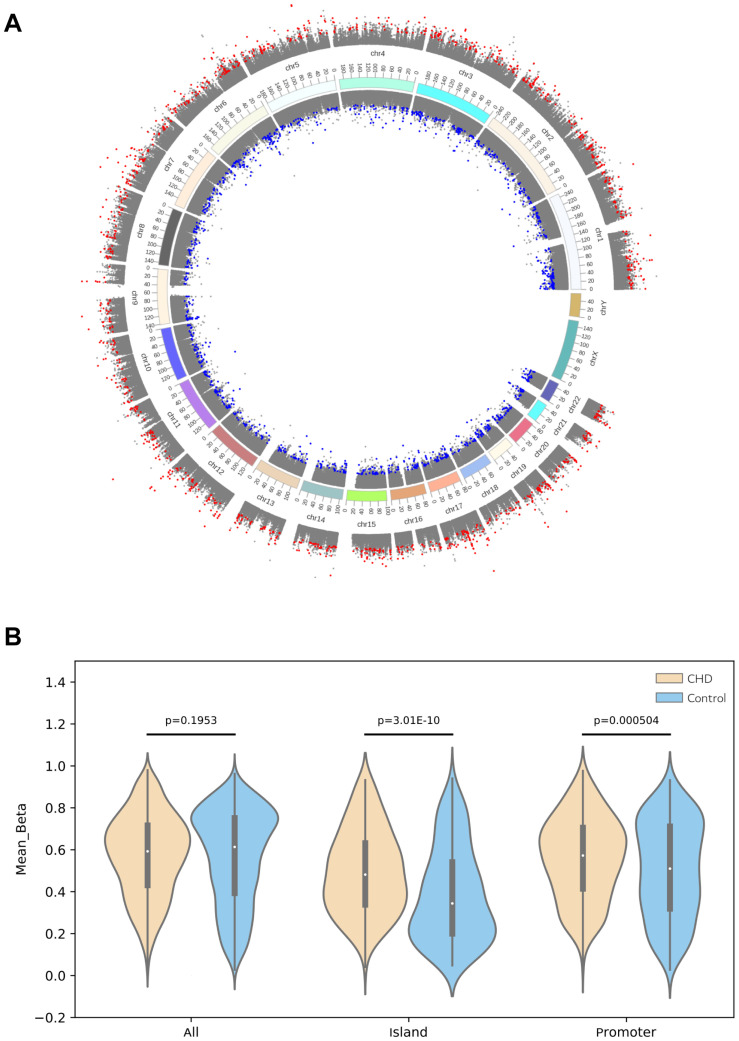
CHD-associated differential methylation sites. **(A)** Differential methylation sites on the genome (UCSC hg19) were depicted, in which the outer circle indicated that the deltaBeta value was above zero and the inner one was less than zero. Gray indicated non-differential methylation sites, red represented hypermethylation sites, while blue denoted hypomethylation sites. **(B)** Violin plot of differentially methylated positions in patients compared with those in the control group. Methylation differences between CHD and the control group were concentrated at CpG islands, particularly at promoters. The CHD group showed a large number of hypermethylation within CpG islands in contrast to those in the control group (*P* < 0.05).

To verify the relationship between SNP and methylation, the case (three TAPVC, three PA, three TOF, and two PDA patients) and control groups (five healthy people) were enrolled for DNA methylation assay using Methylation EPIC (850 K) Bead Chip. The heatmap, presented in Supplementary Figure 1A, displays the differential methylation levels in CHD cases compared with those in the control group. The decrease or increase of methylation levels was also shown in the volcano map (Supplementary Figure 1B), and we found that 50.3% DMS are in hypermethylation and others are in hypomethylation (49.7%) (Supplementary Figure 1C). The map of DMS genomic distribution was also presented in Figure 3A and Supplementary Figure 2. Analysis of the location of CpG sites that were differentially methylated showed that DMS within islands of CHD were extremely likely to be more hypermethylated compared with those in the control group. Further analysis of these DMS within CpG islands showed that this hypermethylation was particularly concentrated around the promoter compared with other locations (Figure 3B).

We divided DMS into several parts, IGR, TSS1500, TSS200, 5′UTR, 1stExon, Body, ExonBnd, and 3′UTR, and analyzed each kind of genes with GO enrichment and KEGG pathway (Supplementary Figure 3A). In this analysis, Fisher’s exact test was used to calculate the significance of enrichment of each term in biological process (BP), cellular component (CC), and molecular function (MF) (Supplementary Figure 3B). For detailed information, we screened all DMS and found a total of 288 terms with *P* value ≤ 0.05 and FDR < 0.05. Also, the top three terms with the lowest *P* value are “homophilic cell adhesion *via* plasma membrane adhesion molecules (TermID:GO:0007156, *P* value: 1e-14), nervous system development (TermID:GO:0007399, *P* value: 3.2e-14), and cell adhesion (TermID:GO:0007155, *P* value: 2.5e-13).”

### Identification of Pathogenic Genes on the Basis of WES Data Analysis and DNA Methylation

Three ways were used to figure out the potential CHD-related genes with differential methylation level to define their potential functional impact. (1) A total of 34 genes were found to show differential methylation changes filtered by Fisher’s exact test and Burden analysis in WES data, of which 22 were hypermethylated and 12 were hypomethylated in SNPs (Supplementary Table 2). (2) Screening WES differential data, we discovered that 180 genes of SNPs were located in the cpG island. (3) According to methylation sequencing and WES differential data, 155 genes with DMS were found to be involved in the mutation-methylation regulations in meQTL database, which mostly play an indispensable role in the pathogenicity of CHD.

**FIGURE 4:**
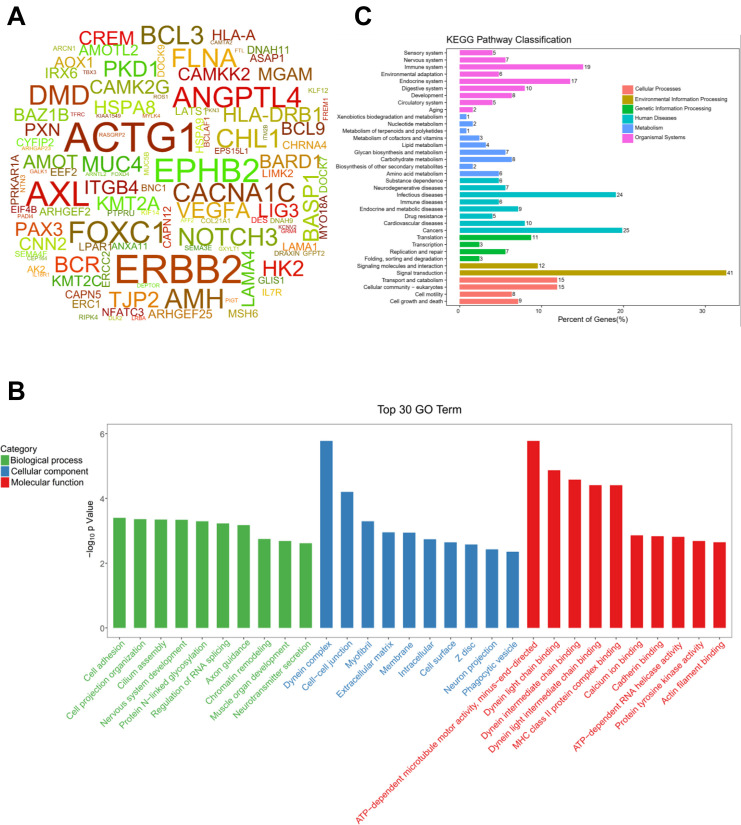
The CHD-associated methylated genes were firstly identified by gene-based burden analysis and Fisher’s exact test and then figured out by three criteria of SNP sites in the cpG island, differential methylation sites, and mutation-methylation regulations in meQTL database, respectively. **(A)** The word cloud of the CHD-associated differential methylated genes derived from above three criteria (*P* < 0.05). **(B)** The top 30 gene terms were identified significantly by SNP sites in the cpG island, differential methylation sites, and mutation-methylation regulations in meQTL database (*P* < 0.05). **(C)** Pathways for the Kyoto Encyclopedia of Genes and Genomes (KEGG) classification.

**FIGURE 5:**
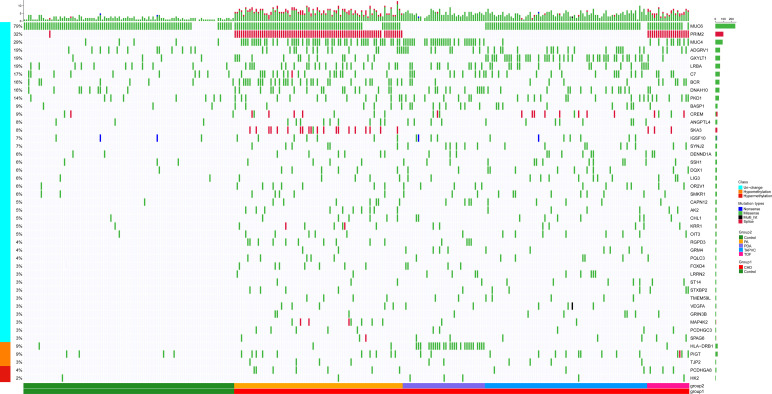
There were 45 methylated genes with higher incidence frequency in patients compared with healthy people through three methods of selection, and the heatmap showed these genes’ distribution in patients. The bars on the right of the panel represented the genes and mutant ratio in the sample, respectively. The color of every box represented the mutation type. Methylation alteration of each gene was also shown. Samples were divided into CHD containing four subtypes (PA, PDA, TAPVC, and TOF) and the control group.

Targeting those above genes, the cloud map was provided for revealing the potential genes with methylation alteration (Figure 4A). GO enrichment analysis of those genes was shown, in which the top three pathways in the BP were “cell adhesion,” “cell projection organization,” and “cilium assembly” (*P* < 0.01, Figure 4B), closely related to cardiac development (Koefoed et al., 2014). Additionally, KEGG enrichment for diseases was related to cancer and infectious and cardiovascular diseases (Figure 4C and Supplementary Figure 4). By assessing gene mutation distribution in different patients, we found 45 damage-associated genes that are much more frequent in patients than in healthy children, figured out from the above genes (Figure 5). The detailed sample distribution and the methylation information were also shown, which revealed more methylation level change in the CHD group.

### Regulatory Network of the CHD Methylated Candidate Genes

**FIGURE 6:**
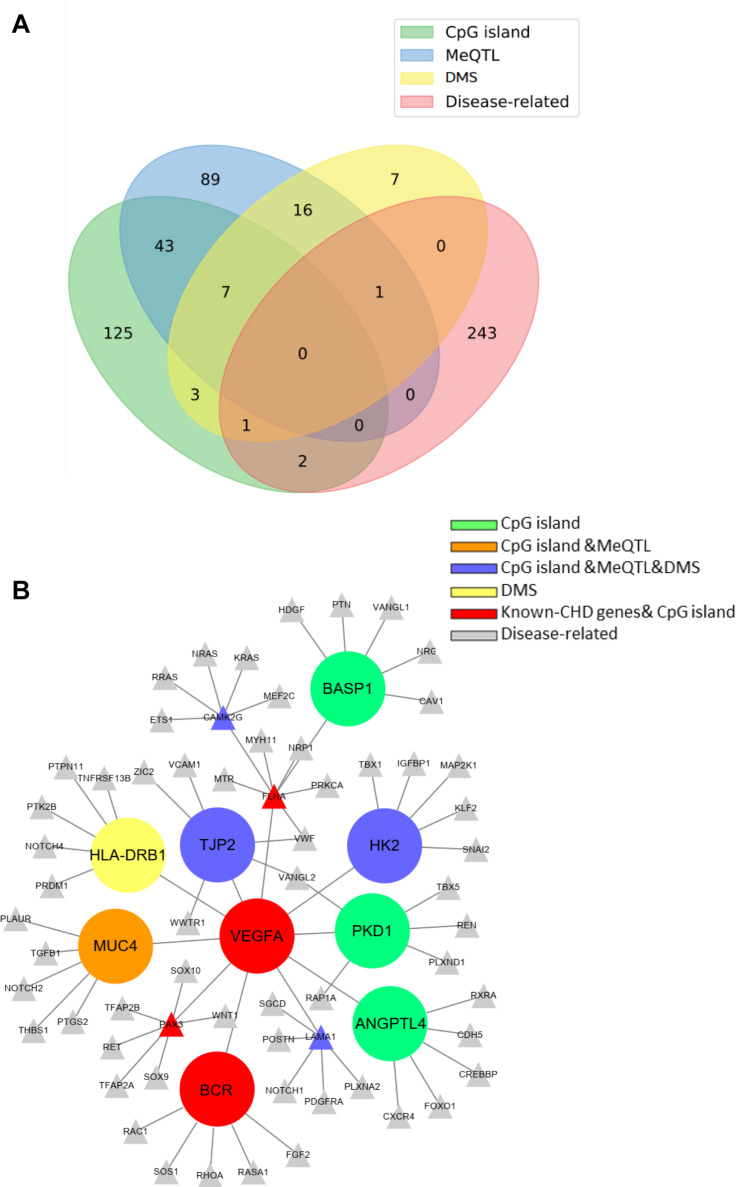
Venn map and regulatory network of the CHD methylated candidate genes. **(A)** Venn map showed the overlap of those genes selected from three criteria. **(B)** The protein–protein interaction (PPI) network of known (gray nodes) and novel CHD-associated methylated genes (The different color represents the candidate genes derived from different screening criteria).

We also collected some known CHD genes that were higher or lower methylation the methylation level in CHD from a large number of reports in Supplementary Material 2. Venn map showed the overlap between genes selected from the three ways and from CHD-related genes, which may contain hyper- or hypomethylated DMS (Figure 6A). Surprisingly, CHD-related genes overlapped with methylated genes selected from any of the above methods, indicating that methylation is very critical for CHD occurrence. Next, we constructed a network composed of known disease-related genes, SNPs that were located in the cpG island, differentially expressed genes with DMSs, and those involved in methylation regulation in database. The shortest links were plotted among the different gene sets with Cytoscape. Finally, nine candidate genes related to methylation were obtained, BASP1, VEGFA, MUC4, HLA-DRB1, TJP2, BCR, ANGPTL4, PKD1, and HK2, which may be involved in the pathogenesis of CHD (Figure 6B) and these genes information was provided in Supplementary Table 3. The mutation sites of BASP1, PKD1, ANGPTL4, MUC4, TJP2, and HK2 were located in CpG islands; MUC4 also was found to regulate the methylation in meQTL data; TJP2, HK2, and HLA-DRB1 were sorted out in WES sequencing with DMS, and the previous two were also involved in SNP-methylation regulation in meQTL data. Moreover, variants of VEGFA and BCR as known CHD genes were also found in the CpG island in our study. The genes’ work indicated that these candidate genes interacted with CHD-related genes and known pathogenic ones. The number of variants of those genes in the case group was higher than those in the control group.

### Mutational Spectrum of Methylated Candidate Genes in the CHD Population

**FIGURE 7:**
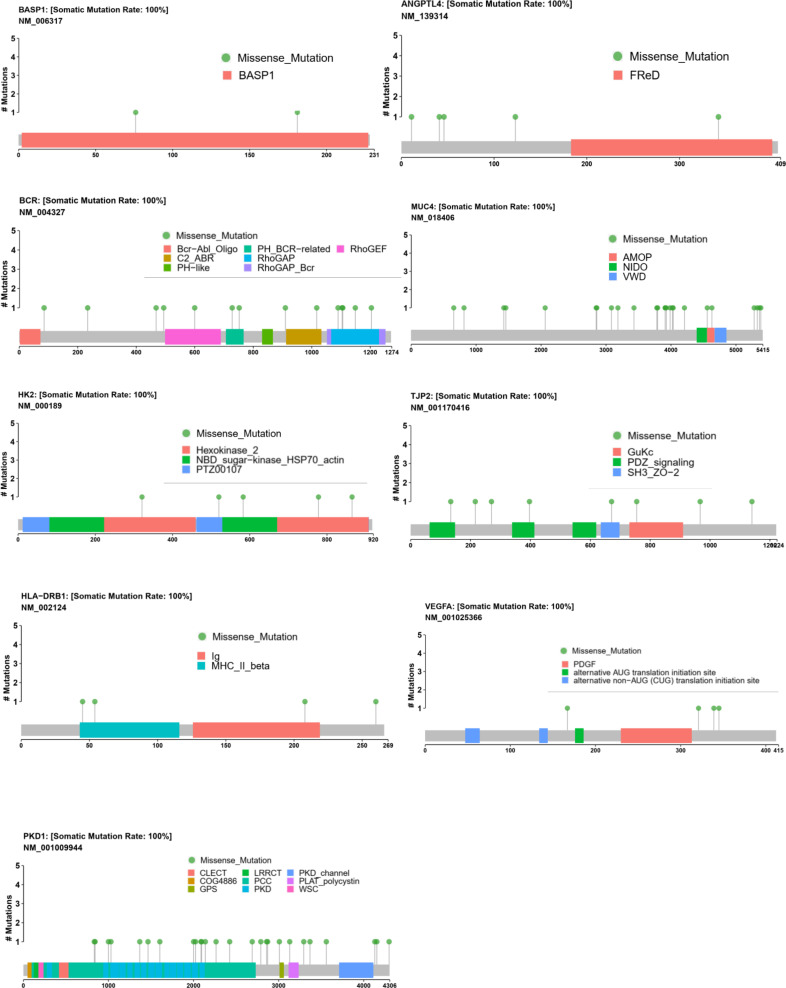
Mutational spectrum of nine methylated candidate CHD genes. The distribution of the candidate genes BASP1, VEGFA, MUC4, HLA-DRB1, TJP2, BCR, ANGPTL4, PKD1, and HK2 in genomics was shown, which displayed the specific amino acid sites of variants of BASP1, BCR, HK2, HLA-DRB1, and PKD1 in the functional protein domains. The mutation sites of ANGPTL4, MUC4, TJP2, and VEGFA were also shown.

When analyzing these genes’ mutational spectrum, we noticed that the non-synonymous variants were mostly located within the functional domains of the coding protein (Figure 7). In particular, all the missense variants of BASP1 and HK2 were located in the BASP1 and HK2 domains separately, which suggested that the two domains were important functional domains and may be closely associated with CHD. Moreover, we also observed variants of HLA-DRB1, PKD1, and BCR, most of which lay in functional domains. Additionally, part of the mutants of ANGPTL4, MUC4, TJP2, and VEGFA were also located in functional domains.

### DMS Level Detection and the Expression Profile in Embryonic Hearts of Nine Candidate Genes

**FIGURE 8:**
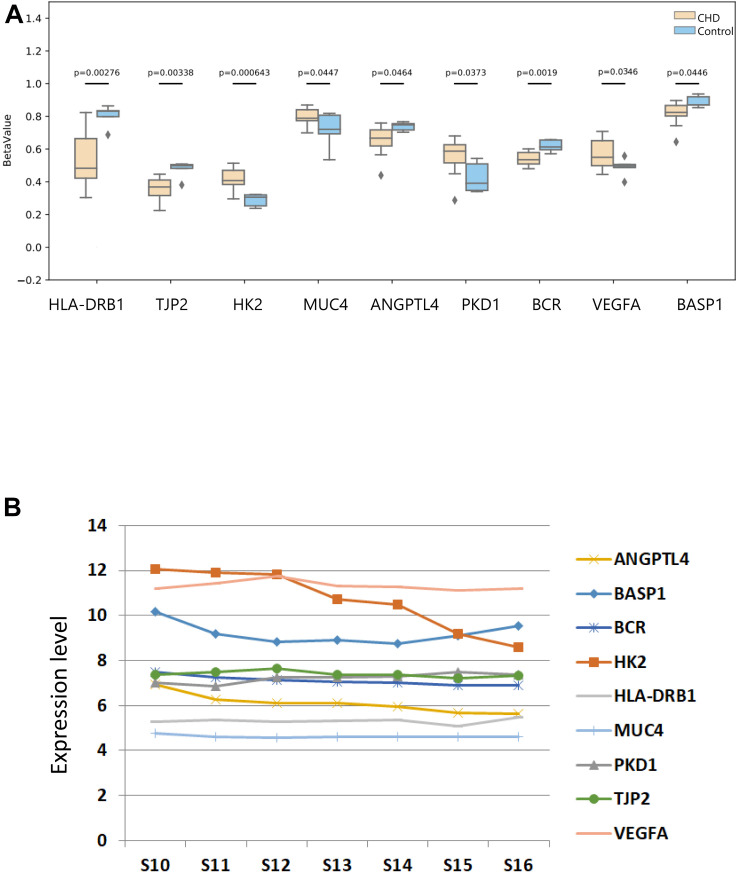
The methylated difference of nine methylated candidate genes in patients and their DNA expression pattern in human embryonic heart. **(A)** The methylated level of nine candidate methylated genes in the case and control groups. The DNA methylation values of sets are depicted with BetaValue (*P* < 0.05). The gray squares mean outliers beyond theoretical expectations statistically. **(B)** The expression patterns of the candidate methylated genes in different development stages of human embryonic heart were provided using an Affymetrix HTA 2.0 microarray.

To investigate the methylation levels of the nine candidate genes, DNA methylation in the methylation chip was observed. Methylation levels of these candidate genes were compared between the CHD and the control group (Figure 8A). HLA-DRB1, TJP2, and HK2 were found, which show DMS directly in our data. MUC4 was screened through meQTL, and the methylated site affected by MUC4 was depicted. ANGPTL4, PKD1, BCR, VEGFA, and BASP1 were selected with the largest absolute value of deltaBeta. The figure depicted that HLA-DRB1, TJP2, ANGPTL4, BCR, and BASP1 showed a lower methylation level in the case group than in the control group with statistical significance, while HK2, MUC4, VEGFA, and PKD1 showed hypermethylation in the case group compared with the control group (*P* < 0.05).

Finally, we then revealed the expression profile of the candidate genes during different stages of embryonic heart development with an Affymetrix HTA 2.0 microarray (Figure 8B). As shown in the figure, the expression levels of HK2, VEGFA, and BASP1 among the pathogenic genes were higher compared with other pathogenic genes in embryonic hearts. In conclusion, we considered ANGPTL4, PKD1, BCR, VEGFA, HLA-DRB1, TJP2, HK2, BCR, and BASP1 as our candidate methylated genes related to CHD.

## Discussion

With the ever-developing technique of WES, many CHD studies based on WES have been published. A large-scale population study could help researchers understand the complex genetics of CHD and identify potential disease-related mutations. Through WES, many *de novo* mutations were predicted to be harmful, causing frameshifts and splicing abnormalities (Zaidi et al., 2013; Gupta, 2013). Gene methylation, as an important epigenetic regulation mechanism, has been widely studied. DNA methylation is the most widely explored epigenetic modification in mammals, which occurs in the context of CpG dinucleotides. In DNA, the cytosine of the CG nucleotides of DNA is selectively added with methyl groups to form 5-methylcytosine, which is commonly found in the 5′-CG-3′ sequence of genes, called CpG dinucleotides (Casati et al., 2015). CpG dinucleotides would be expected to make up ∼4% of a random genome. Studies have found that the methylation of the human genome is a covalent bond modification and occurs at the CpG site (Hartl et al., 2019; Zhi et al., 2013). Genome-wide association studies have revealed genetic loci linked to site-specific of CpGs, named DNA meQTL (Huan et al., 2019; Yao et al., 2018). MeQTL, combined with SNP, was explored for SNP-methylation regulation relationship and applied to a variety of diseases, including cancers. There were studies revealing that *cis* and *trans* CpG-transcript pairs causally were related to cardiovascular diseases. Researchers have identified that linking meQTL variants with disease-associated genetic variants would reveal molecular mechanisms of human diseases in altered epigenetic regulation. However, the interaction of SNP variants and DNA methylation, accompanying the changes of gene expression in CHD occurrence, remains largely unknown.

We documented the methylation regulation and SNP in the DNA of blood. In Group 2, DMS were analyzed and hyper- or hypo-methylation was associated with the expression of the affected genes by *cis*- and *trans*-acting regulation. In this study, we adopted WES and DNA methylation sequencing to identify rare variants and therefore regulated methylation levels of pathogenic genes. Further systematic analysis revealed ANGPTL4, VEGFA, PAX3, MUC4, HLA-DRB1, TJP2, BCR, PKD1, and HK2 as the wholly novel methylated genes related to CHD pathogenesis.

BCR is often found in patients with chronic myelogenous leukemia with a reciprocal translocation between chromosomes 22 and 9. A previous finding reported that BCR-ABL inhibitors have a teratogenic effect on embryo and developmental defects including CHD, hypospadias, and pyloric stenosis (Wang et al., 2017). Furthermore, genetic workup revealed that left ventricular non-compaction (LVNC) in a patient with 22q11.2 distal deletion encompassing the BCR gene (Madan et al., 2010). In our study, BCR was detected in nearly 20% (43/216) of the CHD patients with four different rare harmful variants, and they were all located in the cpG island. We also found that methylation level of BCR was downregulated in patients, which indicated that variants may influence the methylation in heart defects. VEGFA belongs to a member of the PDGF/VEGF growth factor family. This growth factor was explored comprehensively, inducing proliferation and migration of vascular endothelial cells, and disrupting the embryonic blood vessel formation. There were findings suggesting that dysregulated VEGF signaling as a pathogenic mechanism contributing to TOF (Reuter et al., 2019). Luckily, the variants of VEGFA in Group 1 (in patients with PA, TAPVC, and PDA) were also found and were located in the cpG island, which largely proved that VEGFA plays an important role in the formation of CHD and have dysregulation of DNA methylation.

Variants of ANGPTL4, PKD1, and BSP1 were all located in the site of the cpG island, which would explain methylation alteration. ANGPTL4 encodes a secreted glycosylated protein, which contains a C-terminal fibrinogen domain (Oteng et al., 2019). Five rare variants were found amid nine PA patients, nine TAPVC patients, and three PDA patients with a percentage of 10% in our study population. Also, the variations are located in the cpG island, and as noted earlier, the appearance of CpG is positively correlated with DNA methylation levels, which indicated that the variations regulated methylation level. Furthermore, ANGPTL4 was directly observed to link with the subnetwork of the known CHD genes. The encoded protein of ANGPTL4 can function as an apoptosis survival factor for vascular endothelial cells and can inhibit metastasis by preventing vascular growth and invasion. Researchers have found that ANGPTL4 regulated circulating lipids, modulated angiogenesis, and associated diseases, and knockdown of ANGPTL4 reduces atherosclerosis in mice (Aryal et al., 2019; Stitziel et al., 2016). The CHD we explored all contained vascular abnormality in structure; therefore, we speculated that ANGPTL4 may serve as a potential novel candidate gene of CHD with altered methylation. PKD1 encodes a member of the polycystin protein family. It is also involved in cell–cell/matrix interactions, is required for the structural integrity of blood vessels, and regulates G-protein-coupled signal-transduction pathways. It was reported that PKD1-deficient mice displayed abnormal myocardial deformation as well as systolic and diastolic dysfunction (Balbo et al., 2016). Seventeen percent (37/216) of patients were found to carry non-synonymous mutations and the methylation chip revealed PKD1 hypermethylated in patients. BASP1 encodes a membrane binding protein with several transient phosphorylation sites and PEST motifs. We found approximately 11% (11/216) of patients with two SNPs (C > T, p.A76V; C > A, p.P181T). Studies have shown that dysregulated expression and methylation level of BASP1 are directly related to the occurrence and prognosis of cancers, such as hepatocellular carcinoma. Besides, BASP1 plays a tumor suppressive role *in vitro* and was targeted as a treatment in acute myeloid leukemia by promoter methylation (Zhou et al., 2018). Interestingly, the three genes are all linked to CHD-related genes directly, which indicates that they may play a critical role in heart defects by dysregulation of DNA methylation.

MUC4 encodes an integral membrane glycoprotein found on the cell surface. Eight variants were found in 40% (86/216) of patients and the top 1 frequency of variant (rs150551454) was located in chromosome 3. It is reported that MUC4 induced angiogenesis through nuclear translocation of β-catenin in pancreatic cancer (Zhi et al., 2014). We found that MUC4 variation not only existed in cpG island but also had regulation relationship in meQTL. The methylation chip proved that MUC4 was hypermethylated.

HLA-DRB1 presents peptides deriving from extracellular proteins and is related to the immune system. Three variants were caught in 13% (28/216) of patients by Fisher’s exact test and Burden analysis. The variants of HLA-DRB1 were sorted out in WES sequencing with DMS. It participated in the pathogenesis of scleroderma from the view of genomics and epigenomics. HLA-DRB1 was hypomethylated in CHD compared to that in healthy people.

The most important CHD candidate methylated genes, TJP2 and HK2, were identified satisfying all three criteria (cpG island, meQTL, and DMS). TJP2, also named ZO2, acts as a composition of the tight junction barrier in epithelial and endothelial cells and is necessary for correct assembly of tight junctions. The five variants of TJP2 were also located in the cpG island. Additionally, TJP2 was found to regulate DNA methylation in the *cis*-meQTL database. Mutations in TJP2 cause the formation of hepatocellular carcinoma in childhood (Zhou et al., 2015). ZO−2 is down−regulated in a cyanotic patient’s myocardium (Jenkins et al., 2016). HK2 has been invested for angiogenesis in a melanoma *in vitro* assay. Three rare damaging variants of HK2 were discovered in patients, and they were located in the cpG island. Additionally, HK2 regulated methylation level in the *trans*-meQTL database. According to previous studies, HK2 is related to the pathogenesis of cancer and alterations in CpG methylation regulate HK2 expression level (Lu et al., 2019). Microarray data demonstrated that the expression level of HK2 was high in embryonic hearts of different development stages. We speculated that HK2 may be involved in cardiac development. Moreover, the methylation chip proved that TJP2 was hypomethylated and HK2 was hypermethylated. Therefore, these two genes are novel and potential CHD methylated pathogenetic candidates.

Although we clarified nine novel methylated genes through the axis of variation, methylation, and expression involved in CHD etiology, some limitations should be mentioned in this study. First, we need a larger population to validate testing. Second, further studies in animal models and the cellular level are warranted to confirm our findings and reveal how SNPs and methylation contribute to cardiac development. Collectively, our findings highlight that DNA methylation changes may affect gene expression in cardiac development, and we detected nine novel methylation-related genes in CHD patients and showed that variations of those genes may regulate methylation in cardiac development. Our study opens new insights into investigations of CHD pathology in SNP variants and DNA methylation levels.

## Data Availability Statement

The data presented in the study are deposited in the NCBI SRA repository, accession number SRP288538 (SRR12897411–SRR12897449).

## Ethics Statement

The studies involving human participants were reviewed and approved by the Ethics Committee of the Department of Pediatric, Yangpu District Shidong Hospital. Written informed consent to participate in this study was provided by the participants’ legal guardian/next of kin.

## Author Contributions

YY and PZ conceived and designed the project and were responsible for the overall content, YY revised the manuscript. JW contributed to dataanalysis and article writing. XM contributed equally with JW. QZ, YC, and DW collected the clinical samples and information. All authors contributed to the article and approved the submitted version.

## Conflict of Interest

The authors declare that the research was conducted in the absence of any commercial or financial relationships that could be construed as a potential conflict of interest.

## Footnotes

1 http://gong_lab.hzau.edu.cn/Pancan-meQTL/

2 http://www.geneontology.org

3 http://www.kegg.jp/

4 http://string-db.org

## Supplementary Material

Supplementary Figure 1 The drawings showed that the heatmap **(A)**, the volcano map **(B)** and the histogram **(C)** of those genes with differential methylation sites (DMS).

Supplementary Figure 2 The circos of those genes with DMS. From the outer circle to the inside, the chromosome, the SNP position, the gene position, and the position of the CpG island in the genome were shown (*P* < 0.05).

Supplementary Figure 3 GO enrichment and KEGG pathway of those genes with DMS were shown through methylation sequencing in Group 2 using Illumina Infinium Methylation EPIC 850 K Bead Chip.

Supplementary Figure 4 KEGG enrichment of those genes filtered by three criteria. We identified some candidate genes by Fisher’s exact test and Burden analysis, then those methylated genes were figured out by the criteria of the mutation located in the CpG islands of the genome, differential methylation sites and DNA methylation quantitative trait loci (meQTLs) in the database, respectively.
