# Supplementary figures and images for: The Interaction Analysis of SNP Variants and DNA Methylation Identifies Novel Methylated Pathogenesis Genes in Congenital Heart Diseases
Source: Front Cell Dev Biol. 2021 May 4;9:665514. doi: 10.3389/fcell.2021.665514 (PMC8143053; doi:10.3389/fcell.2021.665514)

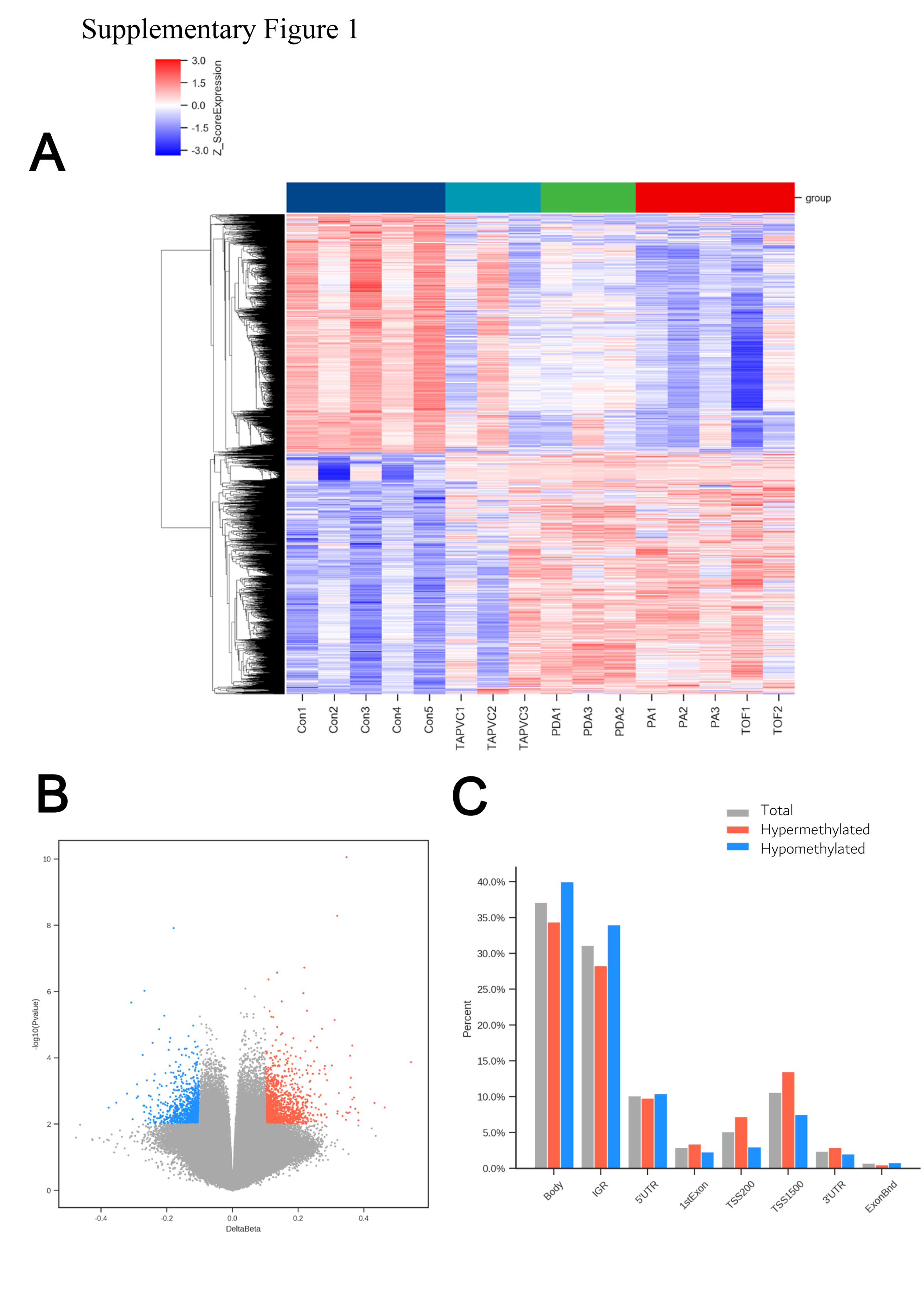

Supplement: Supplementary Figure 1 — The drawings showed that the heatmap (A), the volcano map (B) and the histogram (C) of those genes with differential methylation sites (DMS). [file Figure_1.tif]

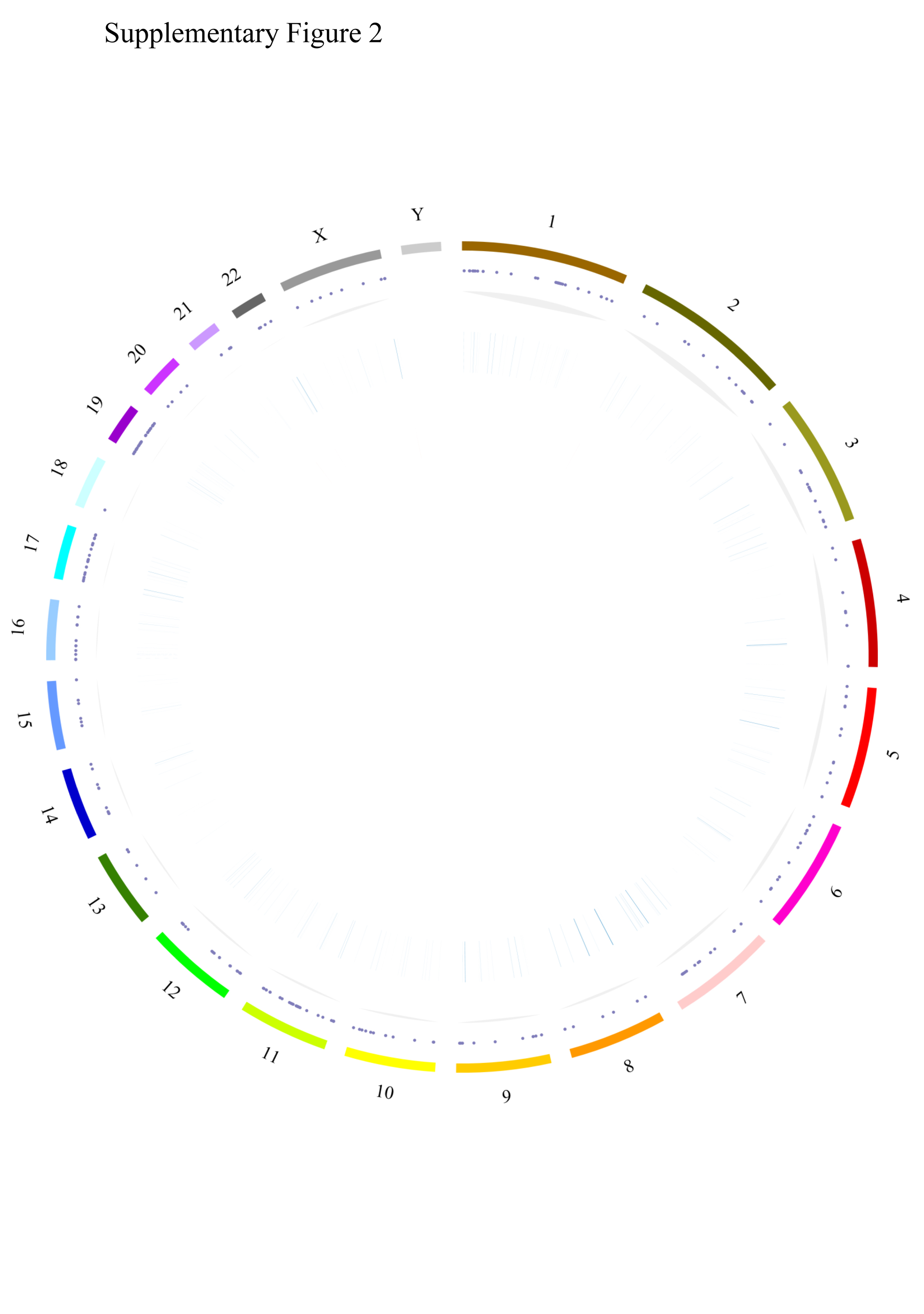

Supplement: Supplementary Figure 2 — The circos of those genes with DMS. From the outer circle to the inside, the chromosome, the SNP position, the gene position, and the position of the CpG island in the genome were shown (P < 0.05). [file Figure_2.tif]

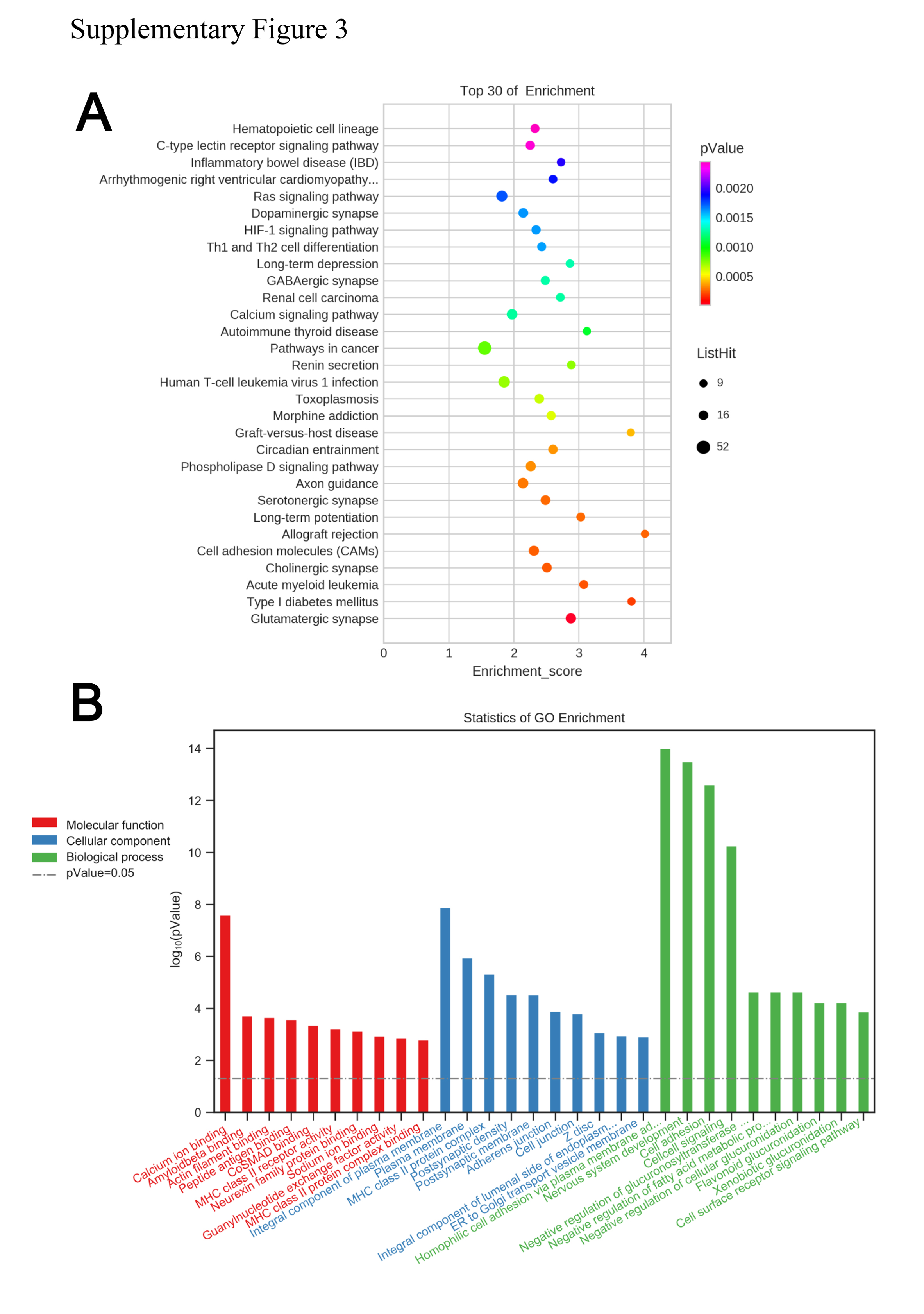

Supplement: Supplementary Figure 3 — GO enrichment and KEGG pathway of those genes with DMS were shown through methylation sequencing in Group 2 using Illumina Infinium Methylation EPIC 850 K Bead Chip. [file Figure_3.tif]

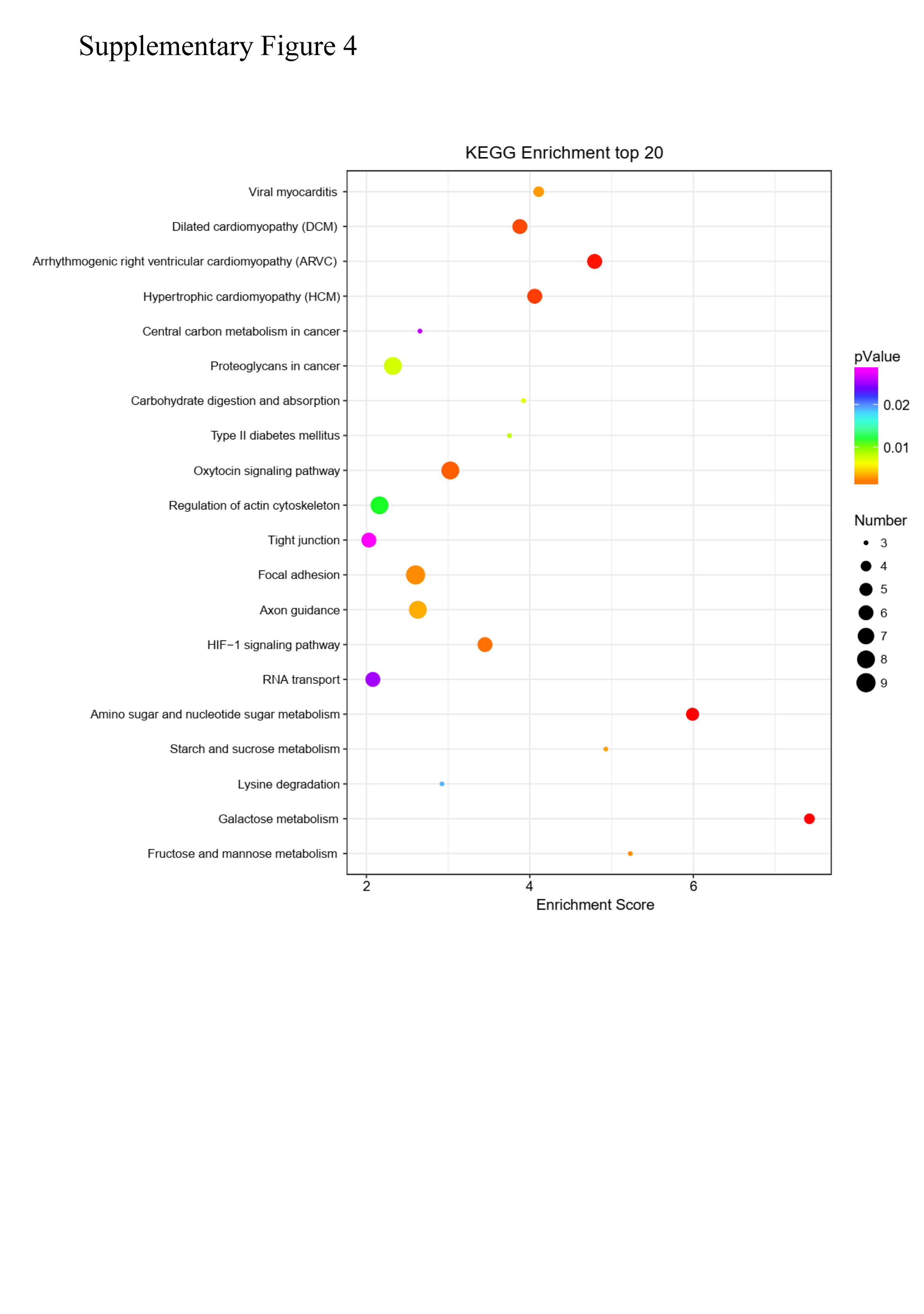

Supplement: Supplementary Figure 4 — KEGG enrichment of those genes filtered by three criteria. We identified some candidate genes by Fisher’s exact test and Burden analysis, then those methylated genes were figured out by the criteria of the mutation located in the CpG islands of the genome, differential methylation sites and DNA methylation quantitative trait loci (meQTLs) in the database, respectively. [file Figure_4.tif]

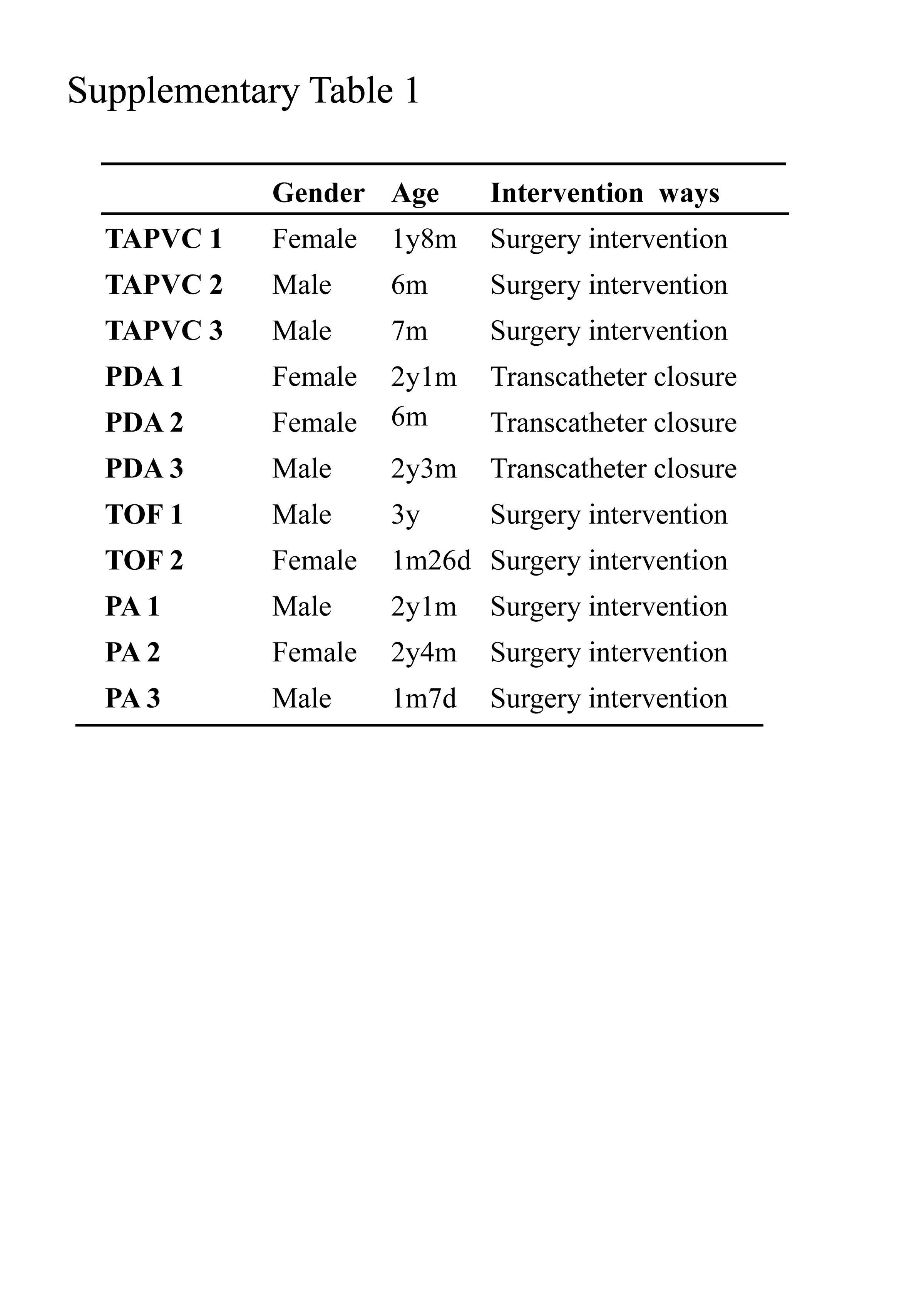

Supplement: Supplementary file 5 [file Data_Sheet_1.zip › Table_1.JPEG]

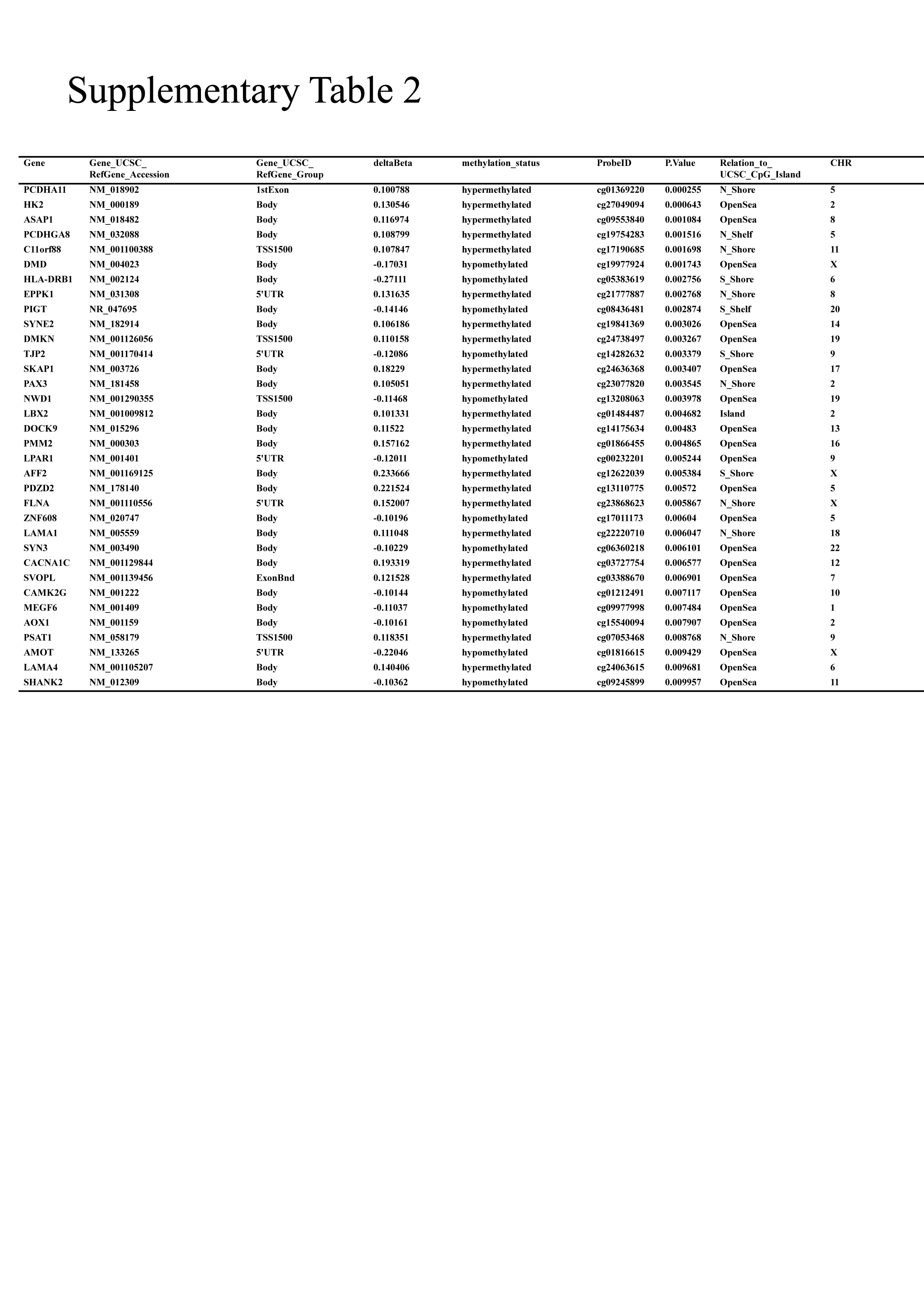

Supplement: Supplementary file 5 [file Data_Sheet_1.zip › Table_2.JPEG]

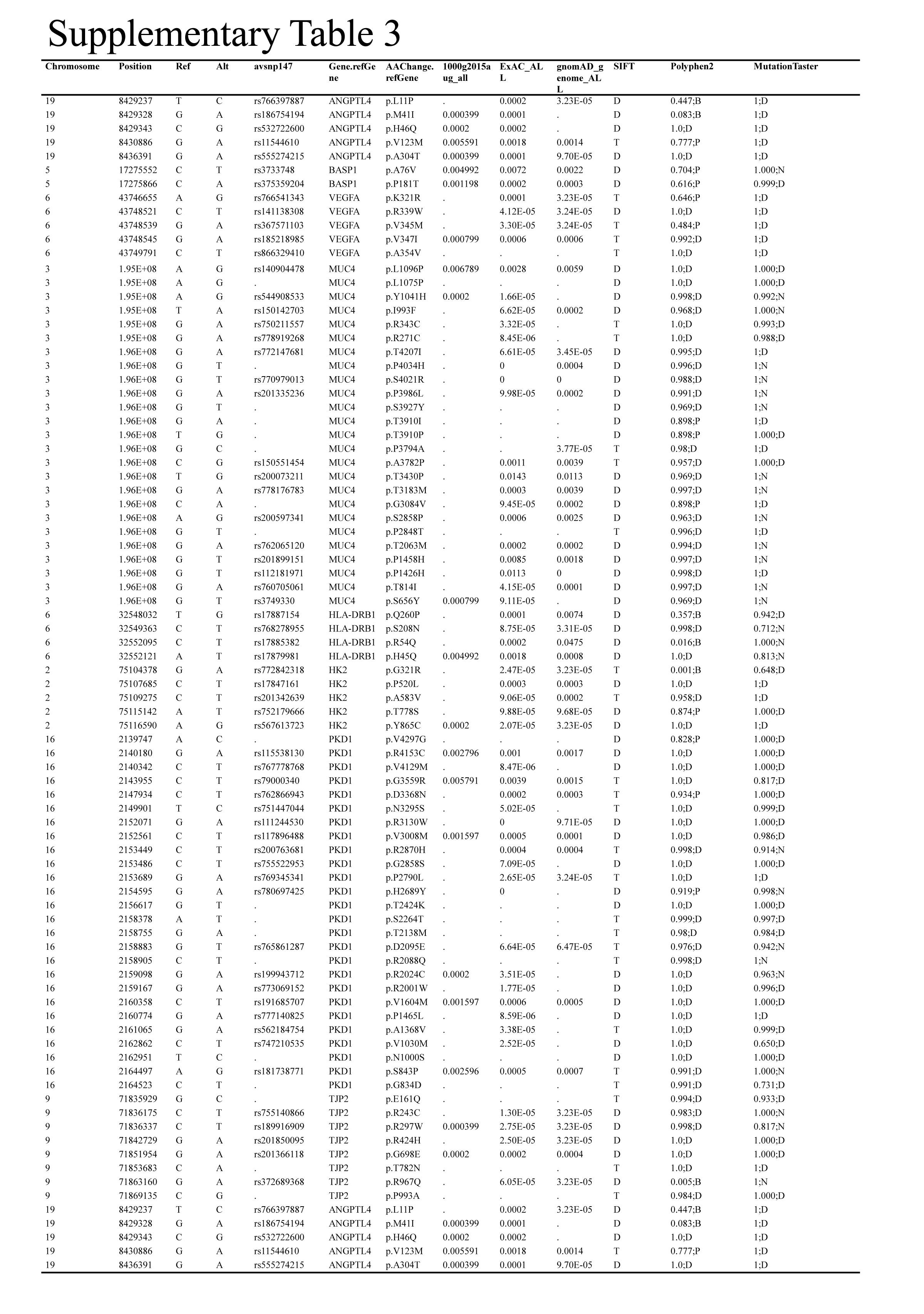

Supplement: Supplementary file 5 [file Data_Sheet_1.zip › Table_3.JPEG]
